# Toward assessing the role of dietary fatty acids in lamb's neurological and cognitive development

**DOI:** 10.3389/fvets.2023.1081141

**Published:** 2023-02-14

**Authors:** Karen Tajonar, Manuel Gonzalez-Ronquillo, Alejandro Relling, Rebecca E. Nordquist, Christian Nawroth, Einar Vargas-Bello-Pérez

**Affiliations:** ^1^Departamento de Medicina y Zootecnia de Rumiantes, Facultad de Medicina Veterinaria y Zootecnia, Universidad Nacional Autónoma de México, Mexico City, Mexico; ^2^Department of Animal Sciences, School of Agriculture, Policy and Development, University of Reading, Reading, United Kingdom; ^3^Facultad de Medicina Veterinaria y Zootecnia, Universidad Autónoma del Estado de México, Toluca, Mexico; ^4^Department of Animal Science, College of Food, Agricultural, and Environmental Sciences, The Ohio State University, Columbus, OH, United States; ^5^Unit Animals in Science and Society, Department Population Health Sciences, Faculty of Veterinary Medicine, Utrecht University, Utrecht, Netherlands; ^6^Institute of Behavioural Physiology, Research Institute for Farm Animal Biology (FBN), Dummerstorf, Germany

**Keywords:** behavior, cognition, DHA, nutrition, welfare, sheep, *Ovis aries*

## Abstract

Understanding and measuring sheep cognition and behavior can provide us with measures to safeguard the welfare of these animals in production systems. Optimal neurological and cognitive development of lambs is important to equip individuals with the ability to better cope with environmental stressors. However, this development can be affected by nutrition with a special role from long-chain fatty acid supply from the dam to the fetus or in lamb's early life. Neurological development in lambs takes place primarily during the first two trimesters of gestation. Through late fetal and early postnatal life, the lamb brain has a high level of cholesterol synthesis. This rate declines rapidly at weaning and remains low throughout adulthood. The main polyunsaturated fatty acids (PUFA) in the brain are ω-6 arachidonic acid and ω-3 docosahexaenoic acid (DHA), which are elements of plasma membranes' phospholipids in neuronal cells. DHA is essential for keeping membrane integrity and is vital for normal development of the central nervous system (CNS), and its insufficiency can damage cerebral functions and the development of cognitive capacities. In sheep, there is evidence that supplying PUFA during gestation or after birth may be beneficial to lamb productive performance and expression of species-specific behaviors. The objective of this perspective is to discuss concepts of ruminant behavior and nutrition and reflect on future research directions that could help to improve our knowledge on how dietary fatty acids (FA) relate to optimal neurological and cognitive development in sheep.

## 1. Introduction

Understanding animal behavior as well as cognitive needs and capacities is needed in modern animal farming, among other reasons to prevent the exposure of farm animals to inadequate welfare conditions (1). Various management practices have been reported to adversely affect welfare in farmed sheep [e.g., (2–6)]. Traditionally, animal cognition has not always been recognized as important to animal welfare at a farm level. This is changing, however, and cognition is currently proposed to be essential for livestock management, as conditions that either improve or are less detrimental to cognitive development, could improve welfare and increase animal's growth (7). Improving our understanding in this field will simplify attempts to adapt husbandry systems and enrichment for farm animals' needs and preferences (1). In humans [i.e., (8, 9)] and rodents [i.e., (10, 11)], factors that impact on cognitive functioning, such as nutrition, have been extensively investigated but this is not the case for farm animals.

We know that in animals, suboptimal neurological and cognitive development can lead to welfare problems due to an individual's potentially impaired ability to cope with its environment (or changes on it). In terms of nutrition, in mammals, dietary fatty acids play a key role on neurodevelopmental functions and ω-3 docosahexaenoic acid (DHA) has been shown to promote synaptogenesis, and neuritogenesis (12), as well as playing important roles during maternal nutrition as it can improve synaptic transmission, and function as a cytosolic signal-transducing factor for gene expression throughout fetal brain development (13).

The objective of this perspective is to discuss basic concepts on behavior and nutrition, and its potential interplay with neurological and cognitive development in sheep. We specially outline future research directions that could help to improve our knowledge on how dietary fatty acids (FA) relate to optimal neurological and cognitive development in sheep. In addition to the role of cognition in farming, sheep are a commonly used animal model for studying gestation and fetal development in humans (14, 15), thus there is translational value of research in sheep lambs for understanding human fetal development.

## 2. Coping with the environment: The role of cognition and stressors

### 2.1. Cognition in sheep

Cognition involves, among other features, learning, memory, attention, and reasoning, which are the combination of internal psychological processes that affect an animal's behavior (16). Learning, remembering, and integrating information helps animals to optimize their decision-making processes in a variety of environmental contexts (17).

For farm animals, an optimal developmental of cognitive traits is key to better cope with their husbandry environment. Studies in sheep have shown that they develop a wide set of cognitive traits to flexibly navigate in their environment [reviewed in (1, 18)].

Sheep are good spatial learners—they learn swiftly how to navigate in a maze task and remember a food location (19). Sheep also easily adapt to a virtual fencing system where they need to associate an acoustic stimulus with an outcome (19). Other cognitive studies on sheep' ability to interpret their physical environment have found that they can make logical inferences in decision-making tasks (20). Learning to flexibly navigate in their husbandry system and making predictions about future events to gain access to crucial resources is key for keeping frustration, and therefore stress levels, low.

Navigating their social environment is also important for sheep. It comes to no surprise that they can discern between different non-human animal species, humans, and figures, which implies high-level skills with involvement of specific neural circuits both in the prefrontal cortex and the temporal cortex (21–23). Likewise, the complex neural processing of visual recognition of individuals related to previous experience that can modify sensory processing has been reported; for example, sheep visually identify other sheep on specific physical characteristics, recognize and memorize conspecifics (23) and humans (24), and are able to differentiate familiar from non-familiar sheep (23). Sheep also form strong mother-offspring bonds which are characterized by the rapid establishment of individual recognition of the lamb using visual and auditory cues (25). These skills are crucial in a husbandry environment but can lead to welfare challenges (e.g., aggression between subjects) when group composition changes or large group sizes limit individual recognition of subjects individuals.

A variety of behavioral and cognitive parameters can be linked to inter-individual differences in behavioral expressions, also often referred to as personality profiles, temperament, coping strategies or coping style (26). Personality profiles are influenced by genetic interaction, age, previous experiences, and environmental conditions, such as the facilities where the animals are kept. Thus, variation of personality profiles should be used for the design and analysis of tests to improve interpretation of behavioral responses (27).

### 2.2. Learning, behavioral flexibility, and coping with the environment

In the wild, animals need to flexibly adapt to their environment to find food and other resources (28). In captive housing environments, animals, too, must adapt to new contexts (e.g., locating and remembering new drinker and feeder positions after transfer to new environments). Their ability to learn, and re-learn new contingencies, here plays a crucial role as subjects who need more time to adapt will experience stress and poorer welfare. Many behaviors shown by farm animals in their daily routine are acquired by learning processes. Learning can take place *via* a variety of mechanisms e.g., through classical conditioning or *via* trial and error (operant conditioning).

Behavioral flexibility refers to the adaptive change in the behavior of an animal, e.g., an animal's ability to learn a now reversed learned contingency and inhibit a previous, not non-rewarded, response. Differences in an animal's ability to learn and/or flexible adapt their behavior, caused by external or internal factors, can be of relevance in the context of various welfare-related issues in farm animals, such as adjusting to new environments or changes in housing and management routines. However, these factors remain relatively unexplored in farm animals.

In the case of sheep, the ability to flexibly adapt to a changing environment could also be linked to specificities of the corresponding production system the animals are kept it (grazing or in confinement). In particular, barren environments can lead to boredom and can increase stereotypies (30–32). The physical husbandry environment can also modify neuronal development and thus cognitive abilities (17). Enriched environments stimulate active, diverse, and flexible behaviors that are desirable compared to barren environments. For example, in goats, environmental and cognitive enrichment has been shown to positively affect goat behavior and learning [e.g., (33)] and could thus also lead to similar effects in sheep. However, environmental, and cognitive enrichment often come with considerable financial costs, as well as with changes in husbandry systems, which makes it, despite its welfare benefits, often not economically viable to be implement in industrial settings.

Learning and flexibility can be compromised in early-life neuronal development [reviewed for humans by (34)], so it is key to identify factors that lead to an optimal development of these traits from early life on.

## 3. Neurological and cognitive development in sheep/lambs

### 3.1. Sheep characteristics

Before revising specifics on neurological development, it is important to consider specific characteristics of sheep. The lamb's brain is relatively well-developed at birth, and sheep are considered as a pre-natal brain developer (35). Compared to rodents, sheep have a high rate of neurogenesis which allows them to be relatively mature and mobile since birth (36). The ewes produce offspring that have fully functional sensory and motor capabilities (37); lamb survival depends on how fast the lamb stands and gets milk from the udder (38). Sheep also possess good spatial learning abilities and memory and form complex social networks when within their groups (39). Social isolation is, thus, a very stressful situation for sheep (40).

### 3.2. Brain development from prepartum to postpartum in lambs—The importance of fatty acids

Structure and organization of the ruminant brain is similar to that of other mammals, and distinctive features of the ruminant brain are the deep depression of the insula, the pronounced gyri in the cortices, the dominant position of the visual and olfactory systems, and the relatively large dimension of the diencephalon (41).

**Figure 1:**
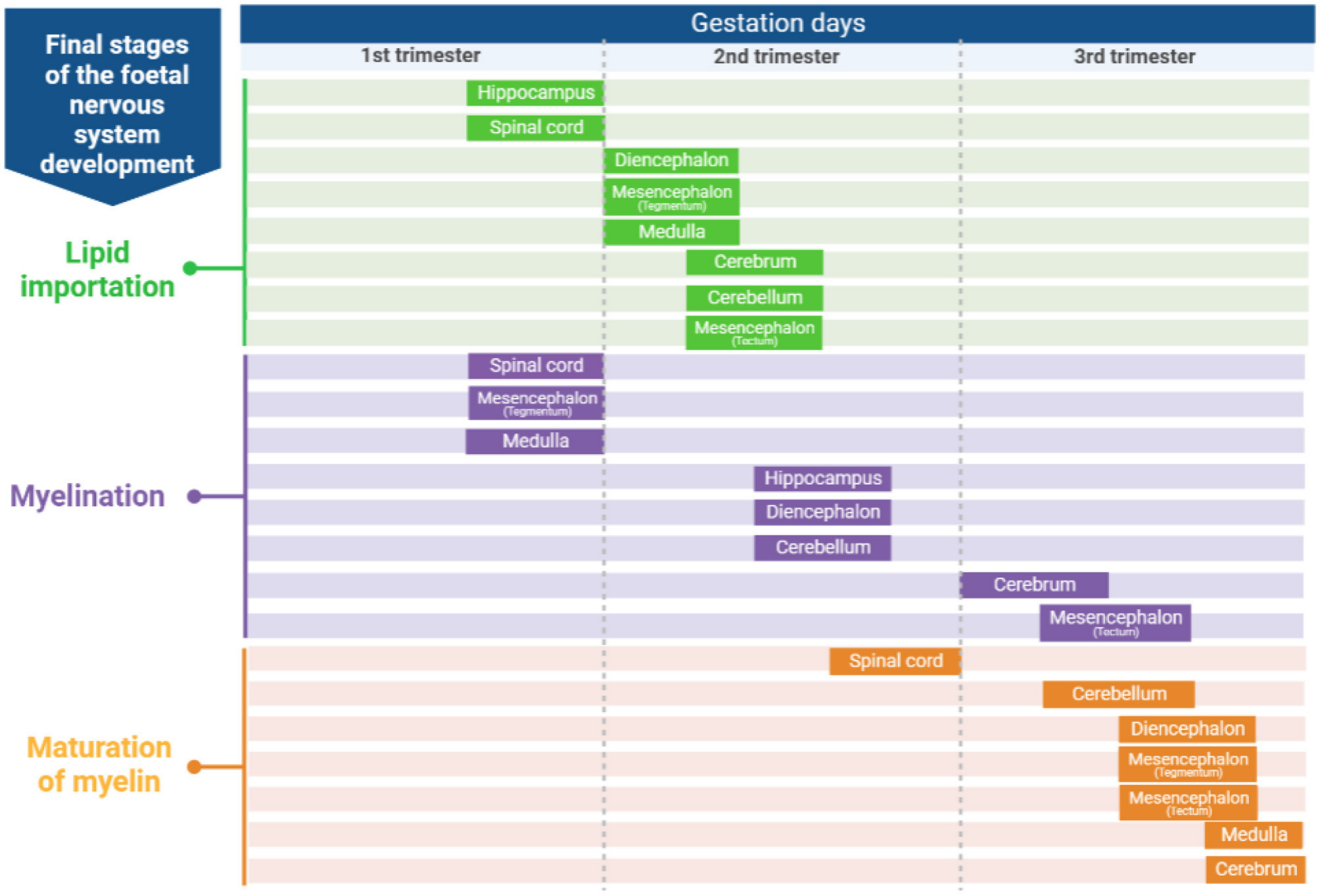
Developmental stages (lipid importation, myelinization and maturation of myelin) of the fetal lamb nervous system [adapted from Barlow (29)]. Created with BioRender.com.

**Figure 2:**
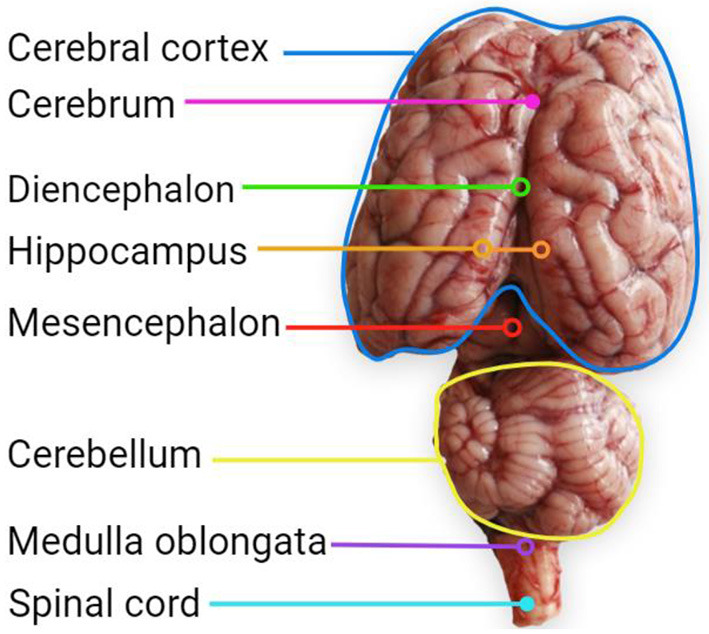
Brain regions of a 3 months weaned female Dorset lamb (89.3 g), with a barley hay-based diet with 5% fishmeal inclusion. 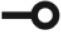 Corresponds to a section that is below the cerebral cortex. Created with BioRender.com.

Knowing how brains develop and the onset of the CNS development stages is important to identify when to perform a more efficient dietary intervention related to dietary FA. According to Barlow (29), in sheep, there are six stages in the development of regions of the CNS (Figure 1): (1) Neuroblast differentiation and migration, (2) Neuronal differentiation, (3) Spongioblast migration and differentiation, (4) Vascular proliferation and lipid importation, (5) Myelination, and (6) Maturation of myelin. Some regions of the sheep brain are shown in Figure 2.

Regarding the role of lipid groups on CNS development, perivascular phospholipids are rapidly integrated for myelin covering whereas galactolipids are gradually used (29). Myelin is critical for smooth electrical signal transmission in neurons and protects neurons against physical forces and offer strong microstructural networks that supports white matter tissue (42).

During the first two-thirds of gestation in fetal lambs, neurological development gradually increases, and connections between the cerebral cortex (the brain structure generally held responsible for higher cognitive functions), and subcortical brain structures (principally the thalamus) lead to the start of sensory perceptions (43). To ensure brain growth and development, through late fetal and early life, the brain has a high level of cholesterol synthesis. This rate declines at weaning and remains low during adulthood (44).

The brain has around 35% lipids consisting of PUFA. They are poorly synthesized in the brain, thus most PUFA come from the liver, and its presence in humans is largely dependent on the intake of fish and other marine products, which is frequently under the advised daily intake (45). For sheep, grasses will be common sources of dietary PUFA (46).

The main brain's PUFA are ω-6 arachidonic acid (ARA) and DHA, which are part of neuronal cell's phospholipids-membranes (47). ARA and DHA are essential nutrients and are important constituents of all cell membranes and are related to membrane fluidity and influence the performance of membrane-bound enzymes and receptors (48–50). DHA is essential for preserving membrane integrity and, therefore, synaptic tasks. Hence, DHA is important for the regular development of the central nervous system (CNS), and its insufficiency damage cerebral activities with irreparable damage (51), resulting in neuropsychiatric disorders (45).

In the sheep fetus, brain weight increases in two phases, before and after 90 days of gestation, and these phases consist of a raise in neuroblast growth accompanied by neuroglial proliferation and myelination. At birth, lamb brain weight is about 50% of the brain weight in an adult sheep (35) (Table 1). Patterson et al. (53) described that there are two peak periods of myelinization related to an increase of cerebroside which occurs about 20 days prior to birth and then at 10–20 days following birth. Myelin is composed by cerebroside which is formed by 24 carbons-PUFA and increase its concentration at 85 days of gestation (Table 2) (53).

**Table 1:** Embryonic and postnatal growth stages in relation to body weight gain, brain weight (absolute and relative to body).

**Stage of development**	**Age days**	**Days of gestation**	**Body WT, kg**	**Brain Wt**		
				**Absolute, g**	**Relative to body wt**, **%**	**Relative adult weight, %** ^*^
	~-110	40	0.00393 ± 0.00065	0.2095 ± 0.0235	6.7	0.20
	~-96	54	0.023 ± 0.002	1.565 ± 0.175	6.7	1.48
	~-83	67	0.080 ± 0.005	4.035 ± 0.545	5	3.83
Early fetal	−73	~77	0.21 ± 0.01	7.1 ± 0.5	3.5 ± 0.1	6.59
	~-69	81	0.25 ± 0.002	9.665 ± 1.065	3.8	9.16
	~-60	90	0.465 ± 0.04	14.27 ± 1.57	3	13.53
	~-55	95	0.640 ± 0.06	19.405 ± 2.135	3	18.40
Midfetal	−42	~108	1.31± 0.12	33.1 ± 4.3	2.6 ± 0.3	30.71
	~-41	109	1.32 ± 0.12	33.675 ± 3.705	2.5	31.93
	~-29	121	2.15 ± 0.14	41.805 ± 4.605	1.9	39.63
Late fetal	−14	~136	3.89 ± 0.23	55.6 ± 1.4	1.3 ± 0.1	51.58
	~0	150		52.74		50.00
Newborn	0	~150	3.83 ± 0.38	53.9 ± 2.7	1.5 ± 0.1	50.00
Suckled	17		9.41 ± 0.25	75.6 ± 1.0	0.8 ± 0.1	70.13
Weaned	105		29.6 ± 1.1	118.2 ± 4.3	0.4 ± 0.1	109.46

Blue: Average live weight, brain weight and percentage brain/live weight ratio of male and female Rambouillet lambs.

Purple: Average live weight, brain weight and percentage brain/live weight ratio of fetuses of female and male Merino lambs [Adapted from Cavender et al., (52); McIntosh et al. (35)].

* Relative brain adult brain weight was calculated as: absolute brain weight for each stage of development × 50 (corresponding to the percentage of the lamb brain weight at birth)/the lambs absolute brain weight at birth corrected for each breed.

**Table 2:** Chemical composition of developing and adult sheep central nervous system divided by cerebrum, cerebellum, brain stem and spinal cord.

	**Stage of development, conceptual age (days)**	**Total lipid (mg/g fresh wt.)**				**Cerebroside (**μ**mol/g fresh wt.)**			
		**Cerebrum**	**Cerebellum**	**Brain stem**	**Spinal cord**	**Cerebrum**	**Cerebellum**	**Brain stem**	**Spinal cord**
Fetal	85	25	23	31	34	1	1	1	3
	110	26	24	45	45	0	2	4	9
	100	33	32	48	64	1	6	11	19
	120	34	39	58	80	1	4	19	33
	130	36	56	77	117	5	11	21	42
	140	50	66	112	132	6	12	35	46
Post-natal	150	53	65	103	150	8	14	31	39
	160	55	71	106	161	10	19	33	40
	170	59	73	113	164	10	18	40	48
	180	66	79	112	191	12	20	37	57
Adult	365	100	93	138	202	25	28	51	78

Mean values ± standard error of the mean.

Adapted from Patterson et al. (53).

The higher the color intensity, the higher the weight.

Fetal programming is the reaction to a specific challenge that a mammalian organism faces during gestation and that modifies the course of fetal development (54). This concept was initially used in human epidemiological data involving low birth weights and inadequate maternal nutrition to an increased prevalence of metabolic disorders (55). Studies connecting fetal programming to animal performance in livestock are relatively recent. These experiments have reported that both under and over nutrition throughout gestation influence offspring growth and performance (56, 57). Consequently, alongside genetics, proper fetal development is needed to reach the growth potential of animals (58), which could be optimized with dietary FA.

In general, in mammals, ω-3 docosahexaenoic acid promotes multiple neurodevelopmental functions, such as synaptogenesis, and neuritogenesis (59). During gestation, dietary FA play an important role for fetal development (13). For example, DHA maintains membrane fluidity, synaptic transmission, and function as a cytosolic signal-transducing factor for gene expression throughout brain development (60). In mammals, compared to ARA (up to 5%), DHA concentration in the neuronal membrane is moderately greater (between 15 and 50% of total FA) (59). While the rapid accumulation of DHA into the brain appears during the last trimester and subsequently during lactation, the maternal DHA concentration must be kept stable through the crucial stage of brain development (59). Therefore, another critical point for brain development is whether lambs are fed on maternal milk or with milk replacers, and in one hand this will be related to the ewes' diet and on the other to the milk replacer formula. In either case, most PUFA are stored in the brain throughout the last third of gestation, which is a period characterized by intense cell division and synaptogenesis (48).

Ruminants have epitheliochorial placentas that are less permeable to free FA than hemochorial (e.g., primates and rodents) (61), and thus, placental transport of short- and long-chain FA in ruminants is restricted (62). Campbell et al. (63) explained that FA in maternal circulation is the primary resource of FA for the fetus and specifically, ω-3 fatty acids are inserted into the placental syncytium by passive diffusion or by membrane-bound carrier proteins and the extent of this transfer will depend on FA affinity to these proteins (50).

Decreased DHA in the developing brain affects neurogenesis, neuro-transmitter metabolism, as well as learning and visual function in animals (48). A lack of DHA through gestation and perinatally cannot be reversed later in life (51, 59). Similarly, Lim et al. (64) conducted a study to establish if supply of preformed dietary docosapentaenoic acid (DPAn-6) could substitute DHA for brain function as evaluated by spatial task performance. In that study, rat pups were fed with DPAn-6 in adulthood and had a lower brain DHA than the dam-reared pups and had negative effects in spatial retention when animals were tested using the Morris water maze. They concluded that DPAn-6 could not replace DHA for brain function, implying a specific structural necessity for DHA.

Taken together, CNS development can be improved by supply of ω-3 FA, and this could be achieved using the fetal developmental programming approach or by supplying these FA during lamb's early life.

### 3.3. Stress and cognitive development

In times of stress, an animal's behavior can become inflexible, hampering their ability to solve problems (65). In sheep, there are events where stress can affect the dam or the offspring, for example, Chronic Maternal Psychosocial Stress during the 1st and 2nd trimester results in extended effects on neuronal network and myelin formation, contributing to disturbed neurobehavioral, cognitive, and motor development in offspring of stressed mothers (66). Neuronal network and myelin formation are pivotal for brain development which as certain proper brain function (66). In ewes, facing stress around the final 3rd of pregnancy increases emotional reactivity and this can be manifested in lamb's deficits in spatial learning (4) and could ultimately affect their production performance.

Cortisol is crucial for the maturation and development of new-borns, and, in sheep, this glucocorticoid cross the placenta during the last third of gestation (67). A natural rise of cortisol appears prior to parturition in at least the last 10 days of gestation in the dam and fetus (68). Extreme cortisol levels of ewes in the last weeks of gestation can affect the fetus and modify placental morphology leading to reduce fetal growth provoked by impaired uteroplacental perfusion (69). Therefore, stressors can affect lamb's behavioral expressions, and this could impede their adaptation to production managements. Avoiding unnecessary stressful situations during sheep gestation can prevent problems on behavior and cognitive abilities in young lambs and this process deserves more research attention.

## 4. Nutrition and its interplay with neurological and cognitive development

Not much information is available on the mechanisms relating milk/dairy intake and cognitive performance (70). Milk from ruminants is characterized by high contents of total saturated FA and low contents of PUFA. This is because several types of ruminal bacteria hydrogenate dietary unsaturated FA and this leads to high contents of palmitic (C16:0) or stearic (C18:0) acids in milk (71). There are differences between ruminant species in terms of milk FA profile (Table 3). Compared to cow's and goat's milk, sheep milk has more milk fat content. Compared to cow's milk, goats and sheep milk have more contents of total saturated FA, specifically those with short- and medium- chain FA (70). Diet and its bioactive components intervene in the development of neuropathologies and it has been reported that saturated fatty acids (SFA) and simple carbohydrates are negative for the brain, while PUFA, polyphenols, and antioxidants are neuroprotective (72, 73).

**Table 3:** Milk fat content (g/100 g) and main fatty acid groups (% of total fatty acids) in milk from ruminants.

	**Cow**	**Sheep**	**Goat**
Total fat	3.3–6.4	4.0–9.0	3.0–7.2
∑ Saturated fatty acids	55.0–73.0	57.0–75.0	59.0–74.0
∑ Monounsaturated fatty acids	2.0–30.0	23.0–39.0	19.0–36.0
∑ Polyunsaturated fatty acids	2.4–6.6	2.6–7.3	2.6–5.6
∑ω-6	1.2–3.0	1.6–3.6	1.9–4.3
∑ω-3	0.3–1.8	0.5–2.3	0.3–1.48

Adapted from Mollica et al. (70).

In sheep, the brain weight is about 0.26% of total body weight (74). In humans, the brain represents around 2% of adult total body weight but spends 20% of the total body's oxygen demand (75). Total cerebral dry weight has up to 50% lipids from which 70% are phospholipids and can be enhanced with DHA (76). Also, DHA has an important role as brain antioxidant (12).

Animals (i.e., rats, young primates, or new-born piglets) with a chronic shortage of dietary ω-3 PUFA, from conception to the early stage of development, exhibit a reduced concentration of DHA in neuron membranes, resulting in retarded visual acuity and impaired learning ability (47, 77). It is thus essential that the fetus receives PUFA through the placenta, and the new-born from maternal milk (47).

Synaptic plasticity supports connectivity between neurons and hence affects the ability of learning and memory through long-term potentiation (LTP). Dietary DHA may stimulate LTP by repairing neurotransmitters release (78). For example, in rats, memory impairment was directly associated with gene expression of presynaptic membrane-associated mediators in the hippocampus and this organ together with the cerebral cortex are the major structures for facilitating memory functions (59). Research in this area is still limited in ruminants.

Neurons and glial cells cannot perform desaturation of FA, such as alpha-linolenic acid (ALA), which is needed for DHA synthesis (79). Therefore, if DHA is needed to be integrated into the brain, it must derive from marine foods or be synthesized in the liver from ALA (79, 80). In sheep diets, oilseeds and their by-products could be sources of ALA and linoleic acid (80, 81). Another milk FA that has been reported to protect against the decline of synaptogenesis (82) and protect cortical neurons from glutamate excitotoxicity in mouse (83) is rumenic acid also known as conjugated linoleic acid (CLA). It has been reported that a maternal supplemented diet with CLA during gestation and lactation results in positive effects on learning and memory in the new-born rats (70, 84). CLA is characteristic of ruminant milk fat, and it is a by-product from a process known as bio hydrogenation where dietary unsaturated FA are chemically changes and many isomers are produced, and CLA is one of them (71, 80).

To our knowledge there is little information concerning how dietary fatty acid supplementation during development affects behavior. Whalin (85) has shown that supplementation of different fatty acids during different stages of life are important for improving learning abilities. In that experiment, ewes were supplemented with either a source of DHA and EPA or saturated (SFA) and monounsaturated fatty acids (MUFA) during early gestation; the offspring was supplemented also with either source of fatty acids, creating 4 groups of lambs (the ones that received always received DHA and EPA, the ones that only received SFA and MUFA, or the ones that received both sources but at different stages of life). On that study there was no difference in the time lambs' needed to solve a maze task; however, those lambs that have received both sources of FA (EPA and DHA) solved the maze much faster the second time than those lambs that had only received one source of fatty acids (85). This result shows the importance of essential fatty acids supplementation during developing; but more research is needed to understand the fatty acid profile required at each specific time of life.

**Figure 3:**
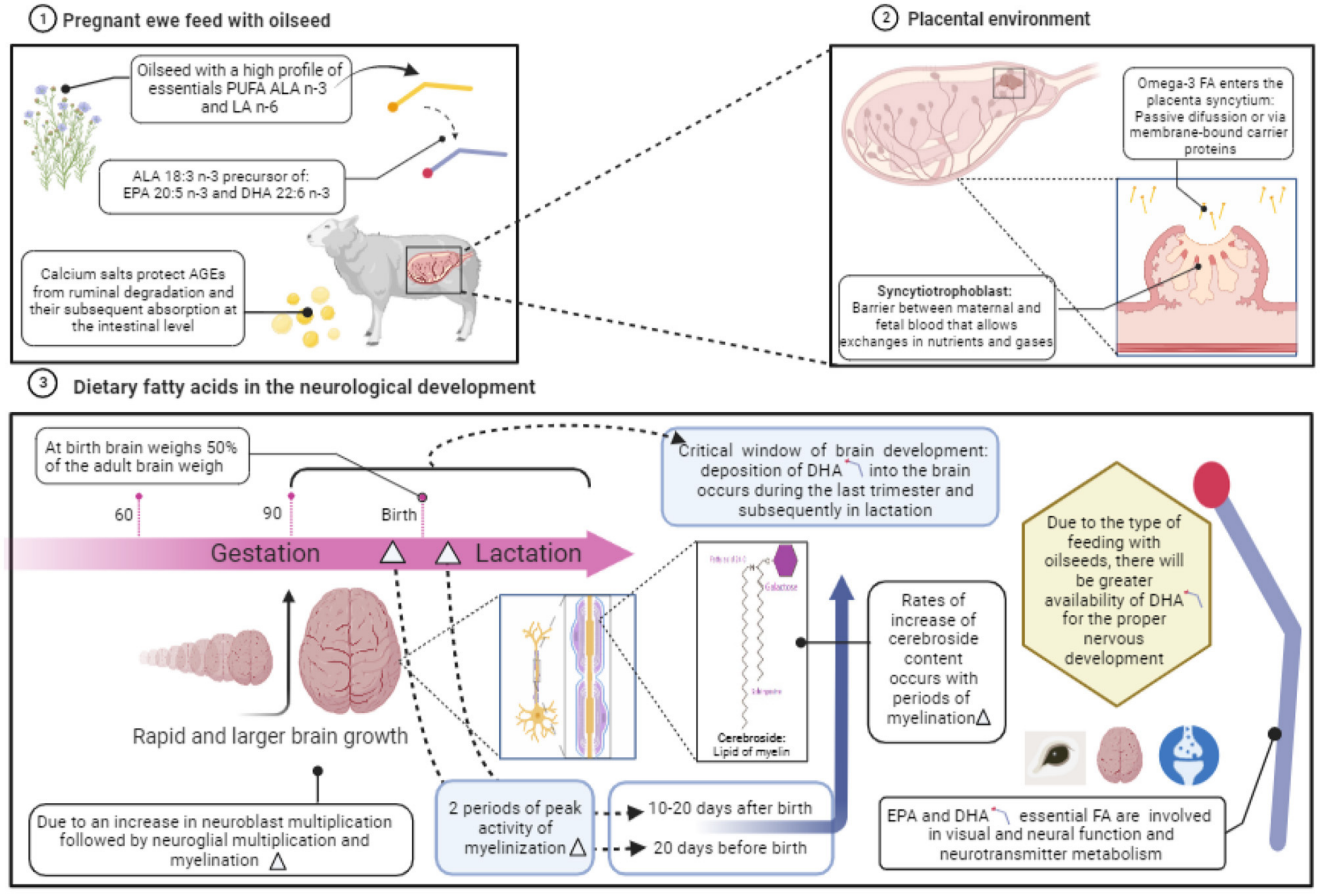
Role of dietary fatty acids on the neurological development during gestation in sheep. Created with BioRender.com.

As discussed, dietary FA can be used as a proxy to improve neurological development in fetus when the dam is on the last trimester of gestation where a rapid brain growth occurs. One feasible option to supply FA to the pregnant ewe is by feeding them with calcium salts of vegetable oils (81). When oils are transformed into salts or soaps, the become rumen inert fats and can increase intestinal absorption of FA which could be transported in the blood until the reach placenta and pass into the fetus (Figure 3).

There are potential pathways on how to investigate the various impacts that nutrition can have on sheep development cognitive development. Most of them have not focused on the interplay between nutrition and neurological and cognitive development. Below maternal conditions and epigenetic factors will be briefly discussed.

### 4.1. Maternal conditions

Maternal environments and nutrition during pregnancy are crucial for fetal development (13). In sheep, maternal nutrition impacts fetus development, for example birth weight can be reduced in lambs born from undernourished dams during second and third thirds of gestation or during whole pregnancy (86). Placental condition is important because throughout the end of the first and beginning of the second trimester of gestation, placental growth happens (87) with the highest growth appearing between days 40 and 60 of gestation (88). Consequently, a maternal dietary restriction during this phase can affect placental development (89) and reduce angiogenesis (86, 90).

Intrauterine growth restriction (IGR) or fetal growth restriction (FGR) refers to infants that failed to reach their *in utero* genetic growth potential leading to low birthweights (14). Intrauterine growth restriction can lead to placental insufficiency and that has an adverse effect on the growth and development of sympathetic nervous system, brain, and heart (15).

In sheep, placental embolization at 120 days of gestation can lead to low birth weight, which relates to reduced myelination, augmented apoptosis and astrogliosis in the cerebral white matter, and reduction of Purkinje cells in the cerebellum, and dendrites growth (91). In this sense it is important to consider that the fetal brain demands that PUFA are provided from the mother through placenta and DHA is the brain's main structural component that is important for membrane fluidity and neuronal signaling (12). Supplementing dams with a source containing DHA have shown to increase the DHA concentration in the fetus brain compared with brains from fetus from non-DHA supplemented dams (92).

### 4.2. Genetics and epigenetics

Performance of individual animals [i.e., rodents (93); goats (94) pigs (95)] in cognitive tests can be related to genetic background. In fish, it has been reported that artificial selection for cognitive traits or brain size can provoke great variations among generations (96). Knowledge on heritability of cognitive traits is complex (17) and in ruminants is a research area that deserves attention.

Polyunsaturated FA regulates gene expression involved in cellular differentiation, growth, and metabolism (97). Omega-3 PUFAs are ligands for transcription factors involved in gene regulation of metabolic and developmental processes (98, 99). Supplementation with ω-3 PUFAs have shown alterations not only in new-born growth (99), but also in mRNA expression on the fetal part of the placenta (92). Also, genes related to lipid metabolism such as free fatty acid receptor, can be affected by maternal supply of ω-3 PUFA (92, 100). Overall, the effect of nutrition and gene expression also known as nutrigenomics deserves further attention as its relationship with animal's behavior and cognition have not been well explored.

## 5. Final remarks and perspectives

**Figure 4:**
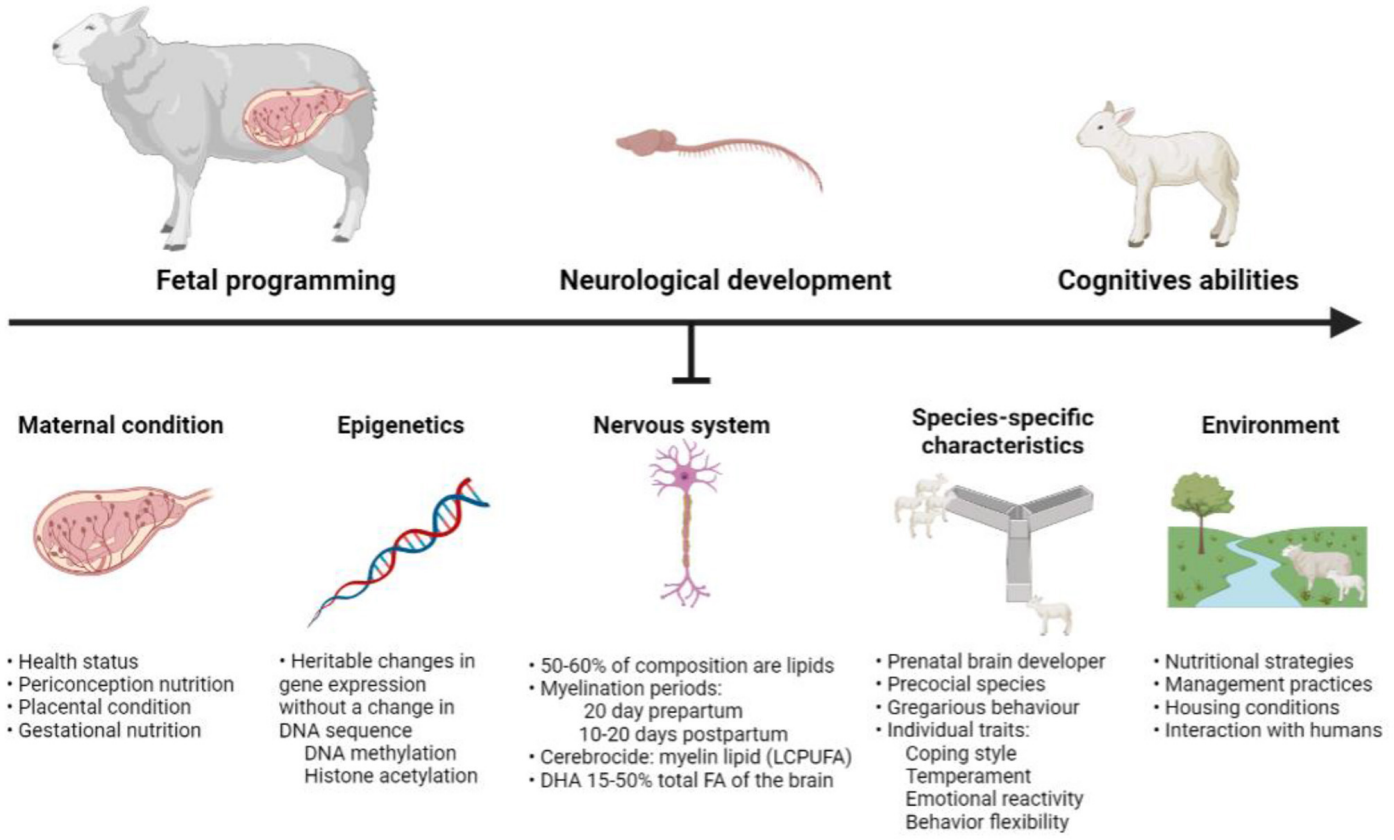
Factors affecting the neurological development of the sheep. Created with BioRender.com.

We here provide a crosstalk between sheep nutrition, cognition and behavior, and many research questions can arise from this outline. As discussed in this manuscript, different factors such as maternal condition (fetal programming) and epigenetics can affect nutritional benefits/supplementation and its subsequent impact on neurological and cognitive development in lambs—with optimally developed individuals being better able to cope with their environment (Figure 4).

An optimal CNS development is crucial for the lamb's life. During gestation, a lack of essential nutrients such as DHA can lead to impairment of lamb's cognitive and behavioral expressions which eventually will affect productive performance. This has been extensively studied in humans [i.e., (101, 102)] but not in sheep. Therefore, we hypothesize that dietary FAs have great potential to improve cognitive development in lambs. Some reports in ruminants suggests that these sources could be derived from marine [products from fish or algae, i.e., (103, 104)] or vegetable [oilseeds and their by-products, i.e., (81, 105)] sources. We further propose that gestation is a critical time to implement a nutritional strategy to improve lamb's cognition (an approach also known as fetal programming); if this is not feasible, we suggested that providing milk and/or other feeds such as oilseeds will stimulate the formation of PUFA that ultimately will reach the brain. We still do not know if a specific type of dietary FA will lead to differential effects on neurological development and cognitive functioning in lambs. We further need to ask whether the most efficient time for a specific nutritional intervention would be before or after birth. An optimal neurological and cognitive development will enable lambs to better cope within the husbandry environment, with positive impacts on their welfare and productive traits.

## Data availability statement

The original contributions presented in the study are included in the article/supplementary material, further inquiries can be directed to the corresponding authors.

## Author contributions

KT, CN, and EV-B-P: conceptualization and visualization. KT and EV-B-P: writing—original draft. KT, MG-R, AR, RN, CN, and EV-B-P: investigation and writing—review and editing. All authors reviewed and approved the final manuscript.
